# Semi-Supervised Fuzzy Clustering with Feature Discrimination

**DOI:** 10.1371/journal.pone.0131160

**Published:** 2015-09-01

**Authors:** Longlong Li, Jonathan M. Garibaldi, Dongjian He, Meili Wang

**Affiliations:** 1 College of Mechanical & Electronic Engineering, Northwest A&F University, Shaanxi, 712100, P.R. China; 2 College of Information Engineering, Shaanxi Polytechnic Institute, Shaanxi, 712000, P.R. China; 3 IMA group, School of Computer Science, University of Nottingham, Nottingham, NG81BB, United Kingdom; 4 College of Information Engineering, Northwest A&F University, Shaanxi, 712100, P.R. China; Ulm University, GERMANY

## Abstract

Semi-supervised clustering algorithms are increasingly employed for discovering hidden structure in data with partially labelled patterns. In order to make the clustering approach useful and acceptable to users, the information provided must be simple, natural and limited in number. To improve recognition capability, we apply an effective feature enhancement procedure to the entire data-set to obtain a single set of features or weights by weighting and discriminating the information provided by the user. By taking pairwise constraints into account, we propose a semi-supervised fuzzy clustering algorithm with feature discrimination (SFFD) incorporating a fully adaptive distance function. Experiments on several standard benchmark data sets demonstrate the effectiveness of the proposed method.

## Introduction

Being one of the most important techniques in pattern recognition, machine learning, data mining and knowledge discovery, clustering is widely used in many application areas to understand and reveal hidden structure of the given patterns. The operation to discover structure is performed by partitioning similar patterns into the same clusters and dissimilar patterns into different clusters based on a certain distance metric, density measure, agglomerative or divisive process, and so on. In general, standard clustering is an unsupervised algorithm which can obtain results that closely match user’s expectations, while classical supervised learning often needs a large number of labelled data to ensure its generalization performance. Semi-supervised clustering integrates the advantages of both, with less human effort, appropriate interaction and adaptable accuracy by taking class labels, prior membership degrees or pairwise constraints into account [1–10].

The research into semi-supervised clustering can be generally divided into two approaches: hard constraints based and fuzzy based methods. In semi-supervised hard c-means clustering methods [1–7], the clustering process is under control of class labels or pairwise constraints to make sure that each instance belongs to only one cluster. Being more specific, hard clustering approaches are crisp methods which are weak in description of the real-world data for the hard binary value of their memberships [11]. In semi-supervised fuzzy c-means clustering methods [8–16], alongside the class labels and membership degrees, pairwise constraints can also be considered to guide the process of the unsupervised clustering and eventually help enhance the accuracy of the algorithm. Recent studies have indicated that the family of fuzzy c-means approaches is a better and more meaningful way to partition the data into groups than hard approaches [10, 12, 13]. In order to overcome the limitations of the existing clustering algorithms, various computational methods with partial supervision have been adopted, ranging from the expectation maximization algorithm for maximum likelihood based parameter estimation [17–19], the integration of an incremental algorithm for the update of classifiers parameters [20], the optimization of an objective or learnable distance function [21], to a classifier retraining to integrate new labelled points [22].

In many situations (when dealing with web document, images, biological information, etc.), the amount of data is too enormous to completely label and pre-processing becomes an essential process to reduce the complexity of the problem. Such pre-processing consists of feature extraction and selection. Feature extraction [19] searches for the smallest possible set of distinguishing or typical features among the feature vectors, whilst the purpose of feature selection [23] is to select and weight the best subset of features from the set of features identified by feature extraction. Most feature weighting and selection approaches are based on the assumption that feature relevance is invariant over real world tasks, and hence a single set of weights is used for the whole dataset. However, feature relevance may vary widely within the domain of a dataset. Following previous work by Frigui [24, 25] and Grira [8], we consider the user’s experiences and the relevance between the feature and prototype centroids in the dataset to guide the process. This requires different feature weights for relevant and irrelevant features; the continuous feature weighting is obtained and the feature relevance representation of each cluster is learned when the clustering is in progress. It is clear that carrying out the clustering and feature selection (weighting) steps simultaneously can speed up the clustering process of the learning system, especially when the constraints provided by users are taken into account.

In this paper, we address the problem of semi-supervised clustering based on both feature discrimination and objective function optimization with an adaptive distance norm. The feature discrimination process attempts to reduce the complexity of the clustering task by eliminating the effect of irrelevant features, whilst the objective function includes two components reflecting the pairwise constraints and feature weights.

The paper is organized as follows. Section 2 outlines the existing algorithms for semi-supervised clustering. Section 3 introduces the algorithm description of semi-supervised fuzzy clustering with feature discrimination. Our experimental setting is described and the results of the comparisons among some semi-supervised algorithms are shown in Section 4. Finally Section 5 contains some conclusions and pointers for future research.

### Related Work

Existing research into semi-supervised clustering has often focused on intensively studying various formulations for constraints, conversion of diverse classical clustering algorithms into partially supervised ones and further discussion about different applications. We generally classify the relevant studies of our proposed method into three categories, namely semi-supervised clustering, semi-supervised fuzzy c-means clustering, and clustering with feature discrimination. In this section, we briefly review some selected examples of existing literature in these categories.

Different approaches can be used to guide the clustering procedure as semi-supervised clustering. In [26], Wagstaff et al. introduced a modified version of clustering with pairwise constraints, namely ‘must-link’ and ‘cannot-link’, to improve clustering performance. Pairwise constraints methods are also solved by using probabilistic models [27], fuzzy clustering models [10], and hierarchical clustering [28, 29]. Later, Basu et al. [30] proposed the k-means algorithm based on seeding to deal with partly labelled data. Then a variant form of fuzzy c-means algorithm based on seeding was proposed by Bensaid and Bezdek [31]. These two approaches refer to the same idea, that is, to calculate simply the mean of the labelled data as seeds to initialize the prototypes of the clusters. Grira et al. [8] proposed an active fuzzy constrained clustering algorithm (AFCC) that minimizes a competitive agglomeration cost function together with fuzzy terms corresponding to pairwise constraints provided by the user.

In addition, since fuzzy c-means (FCM) is one of the most classical algorithms, some related work has been presented as variants of semi-supervised FCM. Yasunori et al. [10] described a semi-supervised clustering algorithm (sSFCM) based on fuzzy c-means clustering by introducing prior membership degree for improving the clustering performance. Pedrycz and Waletzky [21] applied a modified FCM algorithm for considering labelled and unlabelled data of the clustering problems as some augmented objective function. Luis et al. [9] proposed a novel semi-supervised fuzzy c-means algorithm, which employs Gene Ontology annotations as prior knowledge to guide the process of partitioning related genes into groups. Also, kernel-based FCM methods [15, 32] called SSKFCM, which combine semi-supervised learning techniques with the kernel method, were introduced to enhance the fuzzy partition quality. The method extends semi-supervised clustering to a kernel space in order to partition the clusters into groups with nonlinear boundaries in the input space.

Moreover, some efforts have also been made on how to identify and weight the relevant patterns during the whole procedure of the clustering. In Pedrycz, Kira and Wettschereck’s work [33–36], several methods have been proposed for feature selection and weighting to solve the problem of selecting and weighting the best subset of features to upgrade the generalization performance. Furthermore, some effective work has addressed unsupervised feature selection, supervised feature selection and especially semi-supervised feature selection. Unsupervised feature selection [37, 38] evaluates feature relevance by keeping certain properties of the data, while supervised feature selection evaluates correlation between features and class labels. In many real world tasks, such as image retrieval applications [39], semi-supervised feature selection methods [40] especially pairwise constraints [32, 39], are more practical than obtaining the true class labels, because it is easier for us to decide whether some pairs of instances belong to the same class or not.

In this paper, we concentrate on the development of a novel and more effective semi-supervised approach based on an active fuzzy clustering algorithm with few constraints to refine the performance on homogeneous various datasets.

### Semi-Supervised Fuzzy Clustering with Feature Discrimination

As mentioned above, an algorithm called simultaneous clustering and attribute discrimination (SCAD) [24, 25] performs clustering and feature weighting simultaneously to solve unsupervised problems and indicates that, when SCAD is used in conjunction with a supervised learning system, it will offer several advantages. On the other hand, most clustering algorithms generally utilize Euclidean distance to reflect the connection between instances, but this form of distance favours generating clusters of a spherical shape. Such a Euclidean distance performs poorly in practice when each feature of the instance is dependent on others. In this Section, we develop a novel algorithm named semi-supervised fuzzy clustering with feature discrimination (SFFD), attempting to address these issues.

### Model Formulation

The SFFD approach is designed to search for the optimal prototype parameters and the optimal set of feature weights under pairwise constraints. The underlying idea of SFFD is to integrate a fully adaptive distance function, feature weights and pairwise constraints in a unified objective function.

### FCM clustering with adaptive distance norm

According to the Gustafson-Kessel (GK) algorithm, each cluster *i* is allowed to have its own norm-inducing matrix *A*_*i*_, which yields the following inner-product norm in order to detect clusters of different geometrical shapes in one data set, let *d*_*ij*_ be the partial distance between data vector *x*_*j*_ and cluster *i*, we can obtain:

$$d_{ij}^{2}={\left(x_{j}-c_{i}\right)}^{T}\;A_{i}\left(x_{j}-c_{i}\right)$$ (1)

The matrices *A*_*i*_ are used as optimization variables in the function, let *A* denote a c-tuple of the norm-inducing matrices: *A* = (*A*_1_, *A*_2_,…, *A*_*c*_). Let *v*_*ik*_ denote the feature weights of each cluster *i*, *N* denotes the number of samples and *n* stands for the feature number of instances. The objective function of the GK algorithm, additionally weighted by constrained memberships, and is defined by:

$$J=\sum_{i=1}^{C}\sum_{j=1}^{N}u_{ij}^{m}\left(\sum_{k=1}^{n}v_{ik}d_{ik}{}^{2}\right)$$ (2)

Note that the parameter *m* stands for weighting exponent which controls the fuzziness of the clustering algorithm_._ According to relative experiment of *Pal*. *et al* on clustering validity [41], the optimal value of *m* should be chosen between 1.5 and 2.5 based on the research experiences and their median value 2 will be the most appropriate choice when no special preconditions are required. Moreover, some typical semi-supervised clustering algorithms like AFCC [8] and SSKFCM [15] prefer to take 2 as the value of *m*. So the parameter *m* is set to 2.

In Eq 2, *J* can be minimized by simply making *A*_*i*_ less positive definite, that is, *A*_*i*_ must be constrained to avoid that the clusters from uncontrolled growth. The usual way is to constrain the determinant of *A*_*i*_ by allowing it to vary with its determinant fixed corresponds to optimizing the shape of the cluster while its volume keeps constant:

$$\left\|A_{i}\right\|=\rho_{i},\;\rho >0$$ (3)

Using the Lagrange multiplier method, the expression for *A*_*i*_ is obtained as follows:

$$A_{i}={\left(\rho_{i}\;\text{det}(F_{i})\right)}^{\frac{1}{n}}\;F_{i}^{-1}$$ (4)

Where *F*_*i*_ is the fuzzy covariance matrix of the *i* ^th^ cluster defined as:

$$F_{i}=\frac{\sum_{i=1}^{N}{\left(u_{ij}\right)}^{m}\;(x_{j}-c_{i}){\left(x_{j}-c_{i}\right)}^{T}}{\sum_{i=1}^{N}{\left(u_{ij}\right)}^{m}}$$ (5)

Note that Eq 2 describes a generalized squared Mahalanobis distance norm between *x*_*j*_ and the cluster mean *c*_*i*_ and the covariance is weighted by the membership degrees in *U* = {*u*_*ij*_}. This component consists of the first term of SFFD, which allows us to obtain compact clusters. Considering the feature relevance, this term is minimized when only one feature is completely relevant in each cluster, while all the other features are irrelevant.

### Fuzzy clustering with feature discrimination

Feature weight is the key factor in feature discrimination and the constraint on the feature weight can be written down as follows:

$$v_{ik}\in [0,1]\forall i,k;\;\text{and}\;\sum_{k=1}^{n}v_{ik}=1,\forall i.$$ (6)

This constraint must be included as the second term of the augmented objective function. With the value of *m* set to 2, taking the adaptive distance norm of Eq 3 and the feature weight constraint of Eq 6 into account, and applying Lagrange multiplier method, Eq 2 converts into the form:

$$J_{1}=J+\sum_{i=1}^{C}\delta_{i}\sum_{k=1}^{n}v_{ik}^{2}-\sum_{i=1}^{N}\lambda_{i}\left(\sum_{k=1}^{n}v_{ik}-1\right)$$ (7)

Since the rows of *v*_*ik*_ are independent to each other, Eq 7 can be rewritten as the following independent form:

$$J_{1}=J+\delta_{i}\sum_{k=1}^{n}v_{ik}^{2}-\lambda_{I}\left(\sum_{k=1}^{n}v_{ik}-1\right)$$ (8)

Note that *i = 1*,*…*,*C*, where *V*_*i*_ is the *i* ^th^ row of *v*_*ik*_. By setting the derivative of *J*_*1*_ to 0, we obtain:

$$\frac{\partial J_{1}}{\partial \lambda_{i}}=\sum_{k=1}^{n}v_{ik}-1=0$$ (9)

and

$$\frac{\partial J_{1}}{\partial v_{ik}}=\sum_{i=1}^{C}\sum_{j=1}^{N}u_{ij}^{2}\left(\sum_{k=1}^{n}d_{ik}^{2}\right)+2\delta_{i}v_{ik}-\lambda_{I}=0$$ (10)

Then *v*_*ik*_ and *λ*_*i*_ can be obtained as follows:

$$v_{ik}=\frac{1}{n}+\frac{1}{2\delta_{i}}\sum_{j=1}^{N}{\left(u_{ij}\right)}^{2}[\frac{{\left\|x_{j}-c_{i}\right\|}^{2}}{n}-d_{ik}^{2}]$$ (11)

$$\lambda_{I}=2\delta_{i}^{\left(t\right)}-\sum_{i=1}^{C}\sum_{j=1}^{N}{\left(u_{ij}^{\left(t-1\right)}\right)}^{2}\left(\sum_{k=1}^{n}d_{ik}^{2}\right)$$ (12)

It should be noted that *v*_*ik*_ has two parts. The first, 1/n, is the default value if all the features have the same relevance to the cluster. The second part is a bias that reflects the compactness of a feature compared to the others. It could be either positive or negative depending on the choice of *δ*_*i*_, so*δ*_*i*_ can be thought of as a balance between the two parts of *v*_*ik*_. This can be achieved by updating*δ*_*i*_ in iteration *t*:

$$\delta_{i}^{\left(t\right)}=K\frac{\sum_{j=1}^{N}{\left(u_{ij}^{\left(t-1\right)}\right)}^{2}\sum_{k=1}^{n}v_{ik}^{\left(t-1\right)}{\left(d_{ik}^{\left(t-1\right)}\right)}^{2}}{\sum_{k=1}^{n}{\left(v_{ik}^{\left(t-1\right)}\right)}^{2}}$$ (13)

Where *K* is a constant and *u*_*ij*_, *v*_*ik*_, *d*_*ik*_ are denoted by superscript for iteration (*t*-1).

To minimize *J*_*1*_ with respect to the centres *c*_*ik*_, by setting the derivative of *J*_*1*_ to 0, we obtain:

$$\frac{\partial J_{1}}{\partial c_{ik}}=-2\sum_{j=1}^{N}{\left(u_{ij}\right)}^{2}v_{ij}(x_{jk}-c_{ik})A_{i}=0$$ (14)

Reducing the above equation, we get:

$$c_{ik}=\frac{v_{ik}A_{i}\sum_{j=1}^{N}{\left(u_{ij}\right)}^{m}\;x_{ij}}{v_{ik}A_{i}\sum_{j=1}^{N}{\left(u_{ij}\right)}^{m}}=\frac{\sum_{j=1}^{N}{\left(u_{ij}\right)}^{m}\;x_{ij}}{\sum_{j=1}^{N}{\left(u_{ij}\right)}^{m}}$$ (15)

From the view of Eq 14, there are two cases for *c*_*ik*_ depending on the value of the product of *v*_*ik*_ and *A*_*i*_, which is mainly relying on the value of *v*_*ik*_. That is, if the value of *v*_*ik*_ is zero, the value of *c*_*ik*_ will be zero. Otherwise, we use Eq 14 to calculate the value of *c*_*ik*_.

### Taking pairwise constraints into account

As we are aiming for a new search-based semi-supervised algorithm, pairwise constraints are considered, given their wide use in guiding the clustering process towards an appropriate partition. For this purpose, we define an objective function based on Eq 7 with pairwise constraints taken into account. Let *M* denote the set of must-link constraints and *ζ* be the set of cannot-link constraints. Using the fuzzy clustering algorithm described in the previous section, we can rewrite the objective function of SFFD as follows:

$$J_{2}=J_{1}+\alpha \left(\sum_{(x_{i},x_{j})\in M}\sum_{p=1}^{C}\sum_{l=1,l\neq k}^{C}u_{ip}u_{jl}+\sum_{(x_{i},x_{j})\in \zeta }\sum_{p=1}^{C}u_{ip}u_{jp}\right)-\varepsilon_{I}\left(\sum_{k=1}^{C}u_{ik}-1\right)$$ (16)

In Eq 16, the first part is an augmented FCM objective function with fully adaptive distance and feature weights. The second part is pairwise constraint that is weighted by *α*, a constant factor that delineates the relative importance of the supervision. The choice of *α* depends on the relative size of the set of constrained data and unlabelled patterns. Then *α* can be defined as follows:

$$\alpha =\frac{N}{M\prime }\frac{\sum_{k=1}^{C}\sum_{i=1}^{N}{\left(u_{ik}\right)}^{2}d_{ik}{}^{2}}{\sum_{k=1}^{C}\sum_{i=1}^{N}{\left(u_{ik}\right)}^{2}}$$ (17)

where *M′*denotes the number of pairwise constraints.

To minimize *J*_*2*_ with respect to *U* under the constraints, by setting the derivative of *J*_*2*_ to 0, we obtain:

$$\frac{\partial J_{2}}{\partial u_{rs}}=2u_{rs}\sum_{k=1}^{C}v_{rk}d_{rk}^{2}+\alpha \left(\sum_{(x_{r},x_{j})\in M}\sum_{l=1,l\neq s}^{C}u_{jl}+\sum_{(x_{r},x_{j})\in \zeta }u_{js}\right)-\varepsilon_{I}=0$$ (18)

Therefore

$$u_{rs}=\frac{\varepsilon_{I}}{2v_{rk}d_{rk}^{2}}-\frac{\alpha \left(\sum_{(x_{r},x_{j})\in M}\sum_{l=1,l\neq s}^{C}u_{jl}+\sum_{(x_{r},x_{j})\in \zeta }u_{js}\right)}{2v_{rk}d_{rk}^{2}}$$ (19)

By setting the derivative of *J*_*2*_ to 0 with respect to *ε*, we obtain:

$$\frac{\partial J_{2}}{\partial \varepsilon }=\sum_{k=1}^{C}u_{ik}-1=0$$ (20)

So that,

$$\sum_{k=1}^{C}\left(\frac{\varepsilon_{I}}{2v_{rk}d_{rk}^{2}}-\frac{\alpha \left(\sum_{(x_{r},x_{j})\in M}\sum_{l=1,l\neq s}^{C}u_{jl}+\sum_{(x_{r},x_{j})\in \zeta }u_{js}\right)}{2v_{rk}d_{rk}^{2}}\right)=1$$ (21)

$$\varepsilon_{I}=\frac{2}{\sum_{k=1}^{C}\frac{1}{v_{rk}d_{rk}^{2}}}+\alpha \frac{\sum_{k=1}^{C}\frac{\left(\sum_{(x_{r},x_{j})\in M}\sum_{l=1,l\neq s}^{C}u_{jl}+\sum_{(x_{r},x_{j})\in \zeta }u_{js}\right)}{v_{rk}d_{rk}^{2}}}{\sum_{k=1}^{C}\frac{1}{v_{rk}d_{rk}^{2}}}$$ (22)

Substituting Eq 22 in Eq 18, the update equation for the membership values of SFFD can be described as:

$$u_{rs}=\frac{\varepsilon_{I}}{2v_{rk}d_{rk}^{2}}-\frac{\alpha \left(\sum_{(x_{r},x_{j})\in M}\sum_{l=1,l\neq s}^{C}u_{jl}+\sum_{(x_{r},x_{j})\in \zeta }u_{js}\right)}{2v_{rk}d_{rk}^{2}}$$ (23)

Note that the first component in Eq 23 is the membership term of the weighted FCM algorithm with adaptive distance norm, which focuses on weighted distances between feature points and prototypes. The second component considers the available supervision: memberships are reduced gradually by taking the pairwise constraints into account until the optimal values are reached.

### Algorithm Description

The algorithm we propose is based on an iterative search for the optimal prototype parameters and the optimal set of feature weights by locally minimizing the sum of weighted intra-cluster distances while respecting to all the pairwise constraints provided by the user. SFFD updates the relevance weights and partition matrix step by step to reach the optimal result.

After the initialization step, we compute the factor *α* that is used to balance the influence from the constrained data and unlabelled patterns, calculate the adaptive distances, and then *δ*_*i*_, the factor that balances the feature weights. Afterwards, the relevance weights and the partition matrix are updated until the maximum difference in value between the partition matrix in the current iteration and the previous iteration falls below a specified threshold.

**Algorithm1.** SFFD algorithm

Fix the number of clusters *C*;

Initialize the relevance weights *v*_*ik*_ to 1/*n*;

Initialize the fuzzy partition matrix *U*;

Repeat

Calculate the cluster centres *c*_*ik*_ by using Eq 15;
Update *δ*_*i*_ by Eq 13;
Compute *α* using Eq 17;
Compute $d_{ik}^{2}$ for 1≤i≤C and 1≤k≤n;
Update the relevance weights *v*_*ik*_ by using Eq 11;
Update the partition matrix *U* ^(*k*)^ by using Eq 23 and pairwise constraints;

**Until** ‖*U* ^(*k*)^ − *U* ^(*k*−1)^‖ < *ε*

As for most fuzzy algorithms, every instance is assigned to the cluster that has the highest membership. In the end, we check the accuracy of the partition matrix by pairwise constraints. We evaluate the possibility of partitioning the constraints into different clusters and regard the highest possibilities as their clusters. Once the instances belonging to must-links are separated into a different group, we grouped them into one cluster of the highest possibility. If the data items pertaining to cannot-links are grouped into the same cluster, we divide them into two classes.

## Experimental Evaluation

### Methodology and data sets

In order to evaluate our proposed method, we ran a series of experimental studies to evaluate the SFFD algorithm in comparison with several typical clustering algorithms (a traditional unsupervised algorithm and four semi-supervised algorithms). Two popular approaches, *Accuracy* and the normalized mutual information (NMI) measure [42], were utilized to analyse performance during the whole process. Furthermore, a comparison of SFFD without feature weights allowed us to evaluate the effect of feature discrimination on the improvement of the accuracy of classification. Various data sets (see S1 Table) were employed to provide a relatively comprehensive evaluation on the effectiveness of our proposed approach. All the comparisons were performed on data sets (see S3 Table) taken from the UCI-repository (http://archive.ics.uci.edu/ml/). Since various algorithms utilize different information to guide the partitioning process, we use labelled instances to generate pairwise constraints and prior membership for each class as labelled data for each data set. During all the experiment, we set the parameter m as 2 and epsilon as 0.001.

Firstly, we provide accuracy comparisons with FCM, AFCC [8], sSFCM [10] and SSKFCM [15]. FCM is an unsupervised clustering algorithm that represents unsupervised algorithms. SSKFCM is a semi-supervised kernel based fuzzy c-means algorithm using the labelled instances as clustering guide. sSFCM apply the prior membership to complete the process of partition. We also provide comparison with AFCC, which is a typical semi-supervised clustering algorithm relying on pairwise constraints. Since various typical semi-supervised clustering approaches adopt different side information to guide the clustering, we utilized labelled instances to produce pairwise constraints and prior memberships in our experiment.

Secondly, an accuracy comparison between SFFD with weights and SFFD without weights was designed to test the contribution of the weights to the accuracy of classification on four data sets. The same average weight was employed to take the position of weights calculated during the clustering process to eliminate the influence of weight for SFFD without weights. The data sets we choose here have various geometric shapes of clusters (see S1 Fig) for a relatively fair evaluation of the performance of the algorithm.

Finally, NMI comparison among the algorithms mentioned above was carried out on four data sets with various proportions of labelled data to obtain more effective evaluation concerning the clustering quality. Likewise, FCM is used as a baseline and other algorithms as references. NMI is a commonly used method to measure the clustering performance by using the clustering results obtained. A larger value of NMI implies a better clustering performance.

### Evaluation results

For various data sets, 40% of data was randomly selected for each class as labelled data. Each algorithm with labelled data was run 50 times to obtain an average performance with errors. And we provide mean value of their accuracy performances for all algorithms in S2 Table as a base line and the other semi-supervised algorithms for comparisons.

S2 Table implies that as improved FCM algorithms, AFCC, sSFCM, SSKFCM and SFFD can significantly improve the partition accuracy by providing corresponding partial supervision. However, different algorithms result in different clustering accuracy. For example, as a prior membership-based approach, sSFCM utilizes ${\left(u_{i\;k}-{\bar{u}}_{i\;k}\right)}^{m}$ to replace *u*_*i k*_^*m*^ in objective function of FCM. Consequently, its performance outperforms FCM for every data set with the help of side information. For an algorithm with the same pairwise constraints, AFCC just minimizes the sum of intra-cluster distances and neglects the weight for various features. As a result, its capability is weaker than SFFD on all the data sets only except for the *Dermatology* data set. This is attributed to the fact that the feature weights can easily make the points of same clusters closer and those belong to different clusters far away. As S2 Table shows that our SFFD achieves the best performance on nearly all the data sets except for the *Dermatology* data set and all the classify accuracy values of SFFD are above the corresponding mean values on all the data sets. Thus, the SFFD algorithm can produce results that come much closer to our expectations. To visualize the performance promotion that the weights bring to SFFD on accuracy, we use various numbers of constraints to test the effect (see S1 Fig) that the weights have. For every number of constraints chosen, 50 experiments were carried out with randomly selected pairwise constraints to obtain a relatively fair result and to decrease the error at a low level.

In S1 Fig, SFFD with weights results in a better outcome of clustering performance than the algorithm without weights. Especially for *wine* data sets, the best performance of SFFD with weights achieves nearly 7% in the clustering performance compared with SFFD without weights. The result shows the feature discrimination (weights) is a necessary help to partitioning the right cluster into the right group, especially for data sets with a regular shape such as the *waveform* data set and the *wine* data set.

In addition, to obtain a comprehensive evaluation of our study, we change the number of labelled instances during the experiment to get a trend of NMI value on four data sets (see S2 Fig). According to the number of instances of each data set, four series of different labelled data was chosen for performance analysis.

The result in S2 Fig shows that SFFD achieves its best performance as soon as enough instances were labelled according to NMI. For the *Vowel* data set, SFFD improved by more than 10% in performance compared with AFCC with 270 labelled instances. Since SSKFCM is a kernel based method, it obtained a better performance than AFCC, sSFCM on both the *Sonar* data set and the *Wine* data set. Obviously, not only the suitable kernel parameter but also more side information about the data is very important for SSKFCM in the applications. As a pairwise constraint based algorithm, by taking weights into account, SFFD outperforms AFCC on both the *Vowel* data set and the *Wine* data set, while AFCC has a better performance with a relatively less labelled data on the *Scale* and *Sonar* data sets.

From S1 Fig to S2 Fig, the results obtained in terms of accuracy and NMI demonstrates little differences on some data sets. For the *Scale* data set, with respect to accuracy measure, both sSFCM and SSKFCM performs well. However, sSFCM got a better NMI performance than SSKFCM. This implies that more than one evaluation approach is necessary for a comprehensive evaluation on clustering performance. The results show that SFFD can help users improve the classification quality by providing possible constraints.

## Conclusion

In this paper, we have presented a semi-supervised approach that performs clustering and feature weighting simultaneously. Different from the typical algorithms, such as AFCC, SSKFCM and sSFCM, the proposed algorithm SFFD focuses on learning a Mahalanobis distance metric instead of original Euclidean distance during the fuzzy clustering process. Thus, based on the same strategy as existed representive supervised algorithms, SFFD tries to adapt the distances among samples to make the data more separable. With pairwise constraints, SFFD can categorize the partial labelled data by determining the best feature weights within each cluster. Moreover, since the objective function of SFFD is based on that of FCM, it inherits most of the advantages from the FCM family of clustering algorithms. In particular, the proposed SFFD algorithm pays more attention to calculate proper feature weights and make the best use of pairwise constraints to improve the separability of the data. By taking the constraints provided by the user into account, different shapes of data sets can produce results that more closely match our expectations. In future, we shall continually evaluate its performance on other real-world datasets, including image databases, and further investigate how to make it more suitable for real-world clustering applications.

## Supporting Information

S1 Fig Clustering performance variances in accuracy on four different shape data with respect to weight (Variance = SFFD with weights-SFFD without weights). The data sets included *Dermatology* dataset,*Ionosphere* dataset,*Waveform* dataset and *Wine* dataset from UCI-repository. The weights were set to 1/n as average weight for SFFD without weights to compare with the algorithm with normal weight calculated by Eq 11. Variances of classification accuracy between SFFD with weight and SFFD without weights were calculated as variable *V*.
(EPS)

S2 Fig Clustering performance comparison in NMI on four datasets. The data sets included *Scale* dataset, *Sonar* dataset, *Vowel* dataset and *Wine* dataset for NMI evaluations among FCM, AFCC, sSFCM,SSKFCM and SFFD. Various amounts of labelled data were chosen to evaluate the performances for different algorithms.
(EPS)

S1 Table All the data sets used in our experiment. (XLS)

S2 Table Comparison of classify accuracy on eight data sets. (XLS)

S3 Table All the data sets listed in S1 Table. (RAR)
